# Supplementary material for: Genetic Background and Clinical Phenotype in an Italian Cohort with Inherited Arrhythmia Syndromes and Arrhythmogenic Cardiomyopathy (ACM): A Whole-Exome Sequencing Study
Source: Int J Mol Sci. 2025 Jan 30;26(3):1200. doi: 10.3390/ijms26031200 (PMC11818934; doi:10.3390/ijms26031200)
Supplement: Supplementary file 1 [file ijms-26-01200-s001.zip › ijms-3416485-supplementary.pdf]

S1. List of genes associated to sudden arrhythmic death.

ABCA12	AGTR1	CALM1	DMPK	GNAI2	HRC	**LAMA2**	MYL2	RBM20	STX1A	VPS37D
ABCB1	AGTR2	CALM2	DNAJC30	GNB3	IKZF1	LDB3	MYL3	REN	SYNE1	VWF
ABCC6	AGXT2	CALM3	DPP6	GNPTAB	IL18	LDLR	MYOM1	RFC2	SYNE2	WAS
ABCG5	AKAP9	**CASQ2**	DSC2	GP1BA	INA	LDLRAP1	NAV1	RFFL	TBL2	WIPF1
ABCG8	AKT1	CAV3	DSG2	GP6	ITGA2	LEMD2	NCF1	RNF207	TBX3	ZNF365
ABHD11	ALG10B	CLCF1	**DSP**	GPC5	ITGB3	LIG3	NDRG4	RYR2	TBX5	ZNF385B
ABO	ANK2	CLCN1	DTNA	GPD1L	**JUP**	LIMK1	NEBL	SCD	TECRL	ZNF592
ACAD9	ANXA5	CLDN3	EIF4H	GPR37L1	KCNA4	LIN9	NOS1AP	**SCN10A**	TGFB3	
ACADL	AP1G2	CLDN4	ELN	GRIA1	KCND2	LIPC	NOS2	SCN1B	TH	
ACADVL	APOB	CLIP2	EMD	GSTA1	KCND3	LITAF	NOS3	SCN2B	THBS2	
ACE	APOE	COX8A	ENPP1	GSTM1	**KCNE1**	LMNA	NRG1	SCN3B	TKT	
ACE2	ATP2A2	CPB2	ERAL1	GSTP1	KCNE2	LPCAT1	P4HA2	SCN4A	TLR4	
ACSL1	B4GALNT3	CPT1A	ESR1	GSTT1	KCNE3	LRP6	PCSK9	SCN4B	TMEM270	
ACSL3	BAZ1B	CRLF1	EYA4	GTF2I	**KCNH2**	MAF	PGM1	SCN5A	TMEM43	
ACTC1	BAZ2B	CSMD2	F2	GTF2IRD1	KCNJ11	MBL2	**PKP2**	SERPINE1	TNNI3	
ACYP2	BCL7B	CSRP3	F5	GTF2IRD2	KCNJ2	METTL27	PLN	SLC25A45	TNNT2	
ADRA2B	BDKRB2	CTNNA3	FBN1	HAND1	**KCNJ5**	MLXIPL	PON1	SLC27A6	TPM1	
ADRA2C	BID	CXADR	FHL1	HCN4	KCNJ8	MLYCD	**PSEN1**	SLC8A1	**TRIM63**	
ADRB1	BUD23	**DCHS1**	FHOD3	HEY2	KCNQ1	MMP3	PTEN	SLMAP	**TRPM4**	
ADRB2	**CACNA1C**	DEGS2	FKBP1B	HLA-B	KCNQ1OT1	**MYBPC3**	PTPN22	SLN	TTN	
AGPAT3	**CACNA1S**	DES	FKBP6	HLA-DQB1	KCTD1	MYH6	RAB3GAP1	SNTA1	USF1	
AGT	**CACNB2**	**DLG1**	GINS3	HLA-DRB1	KNG1	MYH7	RANGRF	SREBF2	VCL	
